# Decreasing the computing time of approximated reliabilities of genomic estimated breeding values in the single-step genomic best linear unbiased predictor using different core sizes for the algorithm for proven and young

**DOI:** 10.3168/jdsc.2025-0892

**Published:** 2026-01-22

**Authors:** S.N. Sanchez-Sierra, Matias Bermann, Natascha Vukasinovic, Miguel A. Sánchez-Castro, Ignacy Misztal, Daniela Lourenco

**Affiliations:** 1 Department of Animal and Dairy Science, University of Georgia, Athens, GA 30602; 2 Zoetis Genetics and Precision Animal Health, Kalamazoo, MI 49007

## Abstract

The single-step genomic best linear unbiased predictor (ssGBLUP) along with the algorithm for proven and young (APY) are used to compute GEBV in livestock populations with extensive genomic data. Calculating GEBV reliabilities is computationally expensive, particularly with many genotyped animals, because it requires inverting the left-hand side of the mixed model equations. However, reliabilities in ssGBLUP models can be approximated by leveraging the sparse structure of the APY. The primary computational bottleneck of the algorithm lies in a matrix multiplication step, which scales quadratically with the size of the core set. This study aimed to decrease the computing time for approximating GEBV reliabilities in ssGBLUP by reducing the size of the core set in APY without compromising the precision of the reliability approximations. Reliabilities were approximated for a single-trait model for calf respiratory disease in Holsteins (h^2^ = 0.042). A dataset comprising 4,563,070 animals in the pedigree, 1,629,592 genotypes, and 1,585,306 records was used for the study. Core sets of varying sizes (25k, 20k, 15k, 10k, and 5k) were evaluated. Approximated reliabilities obtained with a core set size of 25k were used as a comparison benchmark. Correlations between approximated reliabilities obtained with different core sizes and the benchmark ranged from 0.94 to 1.00, whereas the intercept and slope of the regression of the benchmark reliabilities on the smaller core reliabilities ranged from −0.16 to 0.38 and from 0.64 to 1.15, respectively. Computing times varied significantly, with the fastest approximation (55.02 min) achieved using a 5k core, compared with 171.27 min for the 25k core benchmark. This represents a 3.1-fold reduction in computing time and a 2.1-fold reduction in memory usage when comparing the 25k core size with the 5k core size. Additionally, more substantial savings can be obtained as the number of traits increases. Having fewer genotyped animals in the APY core is a reasonable approach to accelerate GEBV reliability calculations; however, changes in the approximated reliabilities occur, underscoring the trade-off between computational efficiency and the accuracy of the approximations.

## Highlights

• Calculating the reliability of genomic breeding values is computationally challenging.
• We present strategies for reducing computing time when approximating reliabilities in large-scale genomic evaluations.
• Our approach speeds up computations, though reliability approximations may be affected.

## Graphical Abstract

**Figure:**
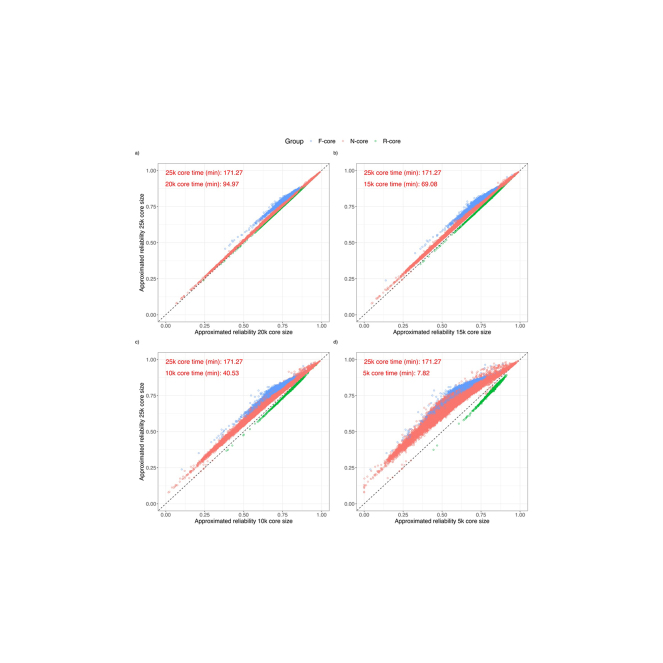
**Summary:** Calculating the reliabilities of genomic breeding values in large datasets is computationally challenging; therefore, approximation methods, such as the algorithm for proven and young (APY) within the single-step genomic best linear unbiased predictor (ssGBLUP) methodology, are used. Reducing the APY core group size from 25,000 to 5,000 genotyped animals decreased the computing time of the reliability-approximation algorithm by up to 95.4%. However, this reduction introduced bias into the approximated reliabilities, highlighting the trade-off between computational efficiency and accuracy.

Genetic evaluation programs provide an estimate of the genetic merit and its reliability, which is a function of the prediction error variance (**PEV**) of the GEBV. When the system of equations is small, the PEV can be derived from the diagonal elements of the inverse of the coefficient matrix of the mixed model equations (**MME**) to obtain the exact reliabilities (Van Vleck, 1993). National genetic evaluations and commercial datasets encompass millions of animals and genotypes, making the direct inversion of the coefficient matrix unfeasible with existing computing resources. Furthermore, including genomic information in the analyses increases the computational complexity of the direct inversion cubically with the number of genotyped animals.

Given these conditions, large-scale genetic evaluations rely on algorithms that approximate the exact reliabilities. While several reliability approximation methods for single-step models are available, they share a common principle in which genomic information contributions are combined with the conventional pedigree contributions through pseudo-observations, resulting in a final estimate that consolidates all available information for both genotyped and nongenotyped animals (Ben Zaabza et al., 2023). Among these steps, including genomic information is the most computationally intensive. It is typically achieved by deregressing genomic reliabilities estimated from either SNPBLUP or GBLUP models. The main advantage of using SNPBLUP models in the step to calculate genomic reliabilities is that the dimension of the coefficient matrix of the MME remains constant with the number of genotyped animals (*n*), making the computing cost dependent on the number of SNPs (*m*), typically fewer than 50k. However, if a residual polygenic effect (**RPG**) is included in the model, the left-hand side must be extended by *n*, increasing the computational complexity from *O*(*m*^3^) to *O*[(*m* + *n*)^3^]. Here, *O*(·) describes the asymptotic computational complexity of an algorithm as a function of problem size. Strategies such as incorporating the RPG in a vector of weights (Gao et al., 2023) or reducing the number of RPG effects using full Monte Carlo sampling techniques (Ben Zaabza et al., 2021) have been developed to alleviate the computational complexity, but not in very large datasets.

On the other hand, GBLUP models are preferred for reliability estimation when *n* is smaller than *m* and because they efficiently account for the RPG. However, their computational cost is *O*(*n*^3^), making them impractical for genetic evaluations involving large numbers of genotyped animals. Misztal et al. (2014a) introduced the algorithm for proven and young (**APY**), which constructs an inexpensive sparse approximation of the inverse of the genomic relationship matrix $\left(G_{\mathrm{APY}}^{-1}\right).$ In APY, the genotyped population is divided into a small group of animals called core set, and a larger group called the noncore set. Taking advantage of the sparse structure of $G_{APY}^{-1},$ Bermann et al. (2022a) developed methods for approximating individual reliabilities in ssGBLUP models with APY. The algorithm is implemented in the BLUPF90 suite (Misztal et al., 2014b) within the ACCF90GS2 software (Bermann et al., 2022c). Unlike other approximation methods, their algorithm does not require setting up the MME to obtain reliabilities of the genotyped animals; instead, it computes a vector of appropriate weights that is added to $G_{APY}^{-1},$ and finally inverts the resulting matrix using block sparse techniques. The most time-consuming tasks in the method are 2 matrix multiplications (steps 4 and 6 of algorithm A1 in Bermann et al., 2022a) with a computing cost that is quadratic with the number of core animals (*n_c_*) and linear with the number of noncore animals (*n_n_*). Therefore, it is desirable to use $G_{APY}^{-1}$ with core groups as small as possible to achieve precise estimates at the lowest computational cost.

Pocrnic et al. (2016) demonstrated that the most accurate GEBV are obtained when the number of genotyped animals equals the number of largest eigenvalues explaining 98% of the variation in the genetic relationship matrix (**GRM**), which in livestock species hardly exceeds 25,000. Core sizes of that dimension may be disadvantageous for estimating reliabilities in large-scale routine genomic evaluations. Moreover, in practical applications, the precision of the approximated reliabilities could be somewhat relaxed because selection decisions are based on GEBV rather than on reliability. Therefore, this study aims to decrease the computing time for approximating GEBV reliabilities in ssGBLUP by reducing the size of the core group in APY without affecting the precision of the approximations.

Phenotypic data for this study were obtained directly from dairy producers upon obtaining their signed permission. Data were provided from approximately 240 herds located in 29 different states in the United States. Two datasets, a complete (Data1_C) and a reduced (Data1_R) one, were used to measure changes in the approximated reliabilities from APY-ssGBLUP when using different core sizes in $G_{APY}^{-1}.$ The complete dataset comprised 4,563,070 animals in the pedigree, 1,629,592 genotyped individuals born from 1975 to 2022, and 1,585,306 records for calf respiratory disease in Holsteins. As described by Gonzalez-Peña et al. (2019), this trait is phenotypically evaluated as a binary response in which a value of 1 indicates that the animal did not experience the disease within the defined time frame, whereas a value of 2 indicates its occurrence. Consistent with their study, we implemented the same animal model and h^2^ = 0.042. The reduced dataset was identical to Data1_C in all aspects except for the number of genotyped animals, which was randomly reduced to 35,000. This design enabled a direct comparison between approximated and exact reliabilities (from direct inversion of MME).

To identify the benchmark core size in $G_{APY}^{-1}$ when approximating reliabilities, we performed a singular value decomposition of the gene content matrix **Z**, associated with Data1_C. We found that 15,095 and 20,852 eigenvalues (square of singular values) explained 98% and 99% of the genetic variance in the GRM, respectively. Considering the core size representing 99% of the variance and rounding it up to the nearest 5k, a core of 25,000 randomly chosen animals was used in this study. This core size not only captured more than 99% of the variance but also aligns with the core size used in Gonzalez-Peña et al. (2019).

To investigate the effect of reducing the core size in $G_{APY}^{-1}$ on reliabilities, we approximated reliabilities using core groups of 25k, 20k, 15k, 10k, and 5k animals across both datasets. First, the core of 25k animals was randomly selected, then smaller core sizes were randomly selected from the next larger core (i.e., animals in $G_{APY}^{-1}$ from the 20k core were selected from the 25k core, 15k from the 20k, and so on; the 5k core animals were retained in all the other larger core groups). We then computed the mean difference (**MD**), Pearson correlation coefficient, intercept, and slope of the linear regression of the benchmark reliabilities from the 25k core on those estimated with each of the 4 smaller core sizes. Changes in reliabilities were assessed only within the genotyped population, which was classified into 3 groups: noncore (**N-core**)—animals consistently classified as noncore across all comparison scenarios; former core (**F-core**)—animals initially included in the benchmark (25k) core group but later reclassified as noncore following core size reductions; and retained core (**R-core**)—animals from the benchmark core group that remained in the core after each reduction.

PREGSF90 (Lourenco et al., 2022) was used to calculate and store all $G_{APY}^{-1}$ matrices. These were later used in BLUPF90+ (Lourenco et al., 2022) to obtain the exact reliabilities in the reduced dataset and the ACCF90GS2 (Bermann et al., 2022c) to approximate reliabilities in each analysis. All analyses were replicated 5 times to obtain the average computing time (wall clock time) and memory usage for each software. The computations were performed on a server with 1.5 TB of memory, 27 TB of disk space, and 2 Intel Xeon 2650 CPUs (2.5 GHz) using 24 cores.

Table 1 presents statistics for the approximated reliabilities for Data1_C across several core sizes in $G_{APY}^{-1}$ for genotyped animals assigned to the F-core, R-core, and N-core groups. Correlations between benchmark and approximated reliabilities exceeded 0.94 across all genotyped animal groups. In addition, MD ranged from 0.00 to 0.14. The intercept and slope of the regression of the benchmark reliability on the approximated reliability with smaller core sizes across all groups of genotyped animals ranged from −0.01 to −0.16 and from 0.64 to 1.15, respectively.

**Table 1:** Correlation, intercept, slope, and mean difference (MD) between 25k-core (benchmark) reliabilities and those from 20k, 15k, 10k, and 5k cores in $G_{\mathrm{APY}}^{-1}$ for a complete dataset (Data1_C)

Analysis	Group^1^	Correlation	Intercept	Slope	MD
20k	R-core	1.00	−0.01	1.01	0.00
	N-core	1.00	0.03	0.97	0.01
	F-core	0.99	0.15	0.85	0.04
15k	R-core	1.00	−0.02	1.02	−0.01
	N-core	1.00	0.07	0.93	0.02
	F-core	0.99	0.19	0.81	0.05
10k	R-core	1.00	−0.05	1.05	−0.01
	N-core	1.00	0.14	0.87	0.04
	F-core	0.98	0.25	0.75	0.07
5k	R-core	1.00	−0.16	1.15	−0.03
	N-core	0.97	0.28	0.74	0.11
	F-core	0.94	0.38	0.64	0.14

1 N-core: animals consistently assigned as noncore across all comparison scenarios; F-core: animals initially part of the benchmark (25k) core group but subsequently reclassified as noncore after core size reductions; R-core: animals from the benchmark core group that remained in the core after each reduction.

Although correlations between the benchmark and approximated reliabilities were high in all scenarios, results showed a consistent pattern: as the core group size decreased, approximated reliabilities deviated more from the benchmark, leading to systematic over- or underestimation across all groups of genotyped animals. These changes in the approximations are expected when reducing the core size in APY because the genomic information in $G_{APY}^{-1}$ is redistributed, altering genetic relationships between core and noncore animals. The extent of these changes is proportional to the variance in the error term for the noncore animals used in the APY formulation. Therefore, the smaller the core size in APY, the larger the variance of the error term and, consequently, the greater the change in the approximations. For more details, we refer the reader to Equations 10 and 14 in Bermann et al. (2022b).

Table 1 shows that the reliability of animals out of the R-core group became increasingly underestimated as the core size decreased, reflected in rising MD and intercept values—particularly for animals in the F-core group. In contrast, reliabilities for animals within the R-core group were slightly overestimated, indicated by decreasing MD and intercept estimates. Overall, the largest deviations in reliability approximation occurred for animals in the F-core group, followed by those in the N-core group. To interpret this behavior, we focus on the comparison statistics from the 20k analysis in Table 1. The theory behind APY is based on conditional distributions. Estimates of the noncore animals are conditioned on the composition of the core group. Hence, when the core group shrank from 25k to 20k, the conditional distributions from the 2 analyses varied proportionally to the size of the reduction. It is worth noticing that the variance of breeding values remains unaffected regardless of the chosen core. Consequently, changes in reliability for the N-core animals are primarily due to the loss of information in the conditional distribution. In contrast, F-core animals experience more pronounced changes, as they shift from being directly connected to the entire genotyped population to only the 20k core group.

We constructed scatter plots comparing benchmark reliability (25k) against reliabilities approximated using smaller core sizes in $G_{APY}^{-1}$ for Data1_C (see Graphical Abstract). We observed that the largest fluctuations in reliability approximations occur among animals with benchmark reliabilities below 0.75. In contrast, animals with higher reliabilities experienced minor changes. This latter group mainly includes cows and bulls with offspring counts ranging from 52 to 13,069. For example, when comparing the 25k and 5k core analyses, animals without progeny or phenotypic records had an MD of 0.13, whereas animals with more than 52 offspring had an MD of just 0.07. In ssGBLUP models, the **H** matrix integrates pedigree and genomic information, providing more precise relationships among nongenotyped and genotyped individuals and enhancing connectivity across families. Changes in reliabilities are more drastic for animals with few progenies and phenotypic records because the accuracy of their GEBV relies mainly on the contribution of genomic information. This aligns with the findings of Brzáková et al. (2023), who measured the impact of incorporating genomic information on the evaluation of age at first calving in Charolais cattle. They found an increase in reliability from BLUP to GBLUP of 0.27 for young bulls (from 0.18 to 0.45), whereas for proven bulls, the increase was smaller, at 0.19 (from 0.35 to 0.54). Their findings underscore the significant impact of genomic information on the reliability of breeding values for animals with limited progeny and phenotypic records.

Other possible strategies for reducing the computing cost of the analyzed algorithm include low-rank approximation methods, such as QR decomposition (Gu and Eisenstat, 1996) and randomized matrix multiplication algorithms (Sarlos, 2006). These methods are especially helpful for accelerating demanding operations like the matrix multiplications in steps 4 and 6 of algorithm A1 in Bermann et al. (2022a). Randomized matrix multiplication decreases the complexity of large matrix products from $\left(n_{n}n_{c}^{2}\right)$ to $O\left(n_{n}n_{c}+n_{c}k\right),$ where *k* is the number of random projections and typically $k<n_{n\;},\;n_{c}.\;$ This is achieved by projecting the original matrices into a lower-dimensional randomized subspace while retaining most of the informative variance. To test this strategy, we implemented a randomized matrix multiplication algorithm in the ACCF90GS2 software, testing values of *k* from 50% to 99% of *n_n_*. However, the approximate reliabilities obtained in all analyses were severely disconnected from the benchmark reliabilities (results not shown).

Figure 1 illustrates changes in computing requirements when approximating reliabilities using various core sizes for $G_{APY}^{-1}$ in Data1_C. The benchmark analysis with a core size of 25k does not include the time (315.8 min) and memory usage (329.79 GB) for computing $G_{APY}^{-1},$ as this matrix is created and stored beforehand during the breeding values estimation. For all other core sizes, these requirements are included. The benchmark computing time (memory use) was 171.27 min (305.29 GB), whereas using the smallest core size reduced the time to 55.02 min (142.82 GB), with all runs using 24 threads. Computing savings in Data1_C were achieved only when the core size was reduced below 15k. These reductions resulted in time savings of 116.25 min and 162.47 GB of memory for the 5k core analysis and 25.14 min and 39.34 GB for the 10k analysis. However, these savings came at the expense of reduced precision in the approximated reliabilities. The computing time solely for the reliability approximation algorithm, excluding the time to compute $G_{APY}^{-1}$ with core sizes of 20k, 15k, 10k, and 5k was 94.97, 69.08, 40.53, and 7.82 min, respectively.

**Figure 1:**
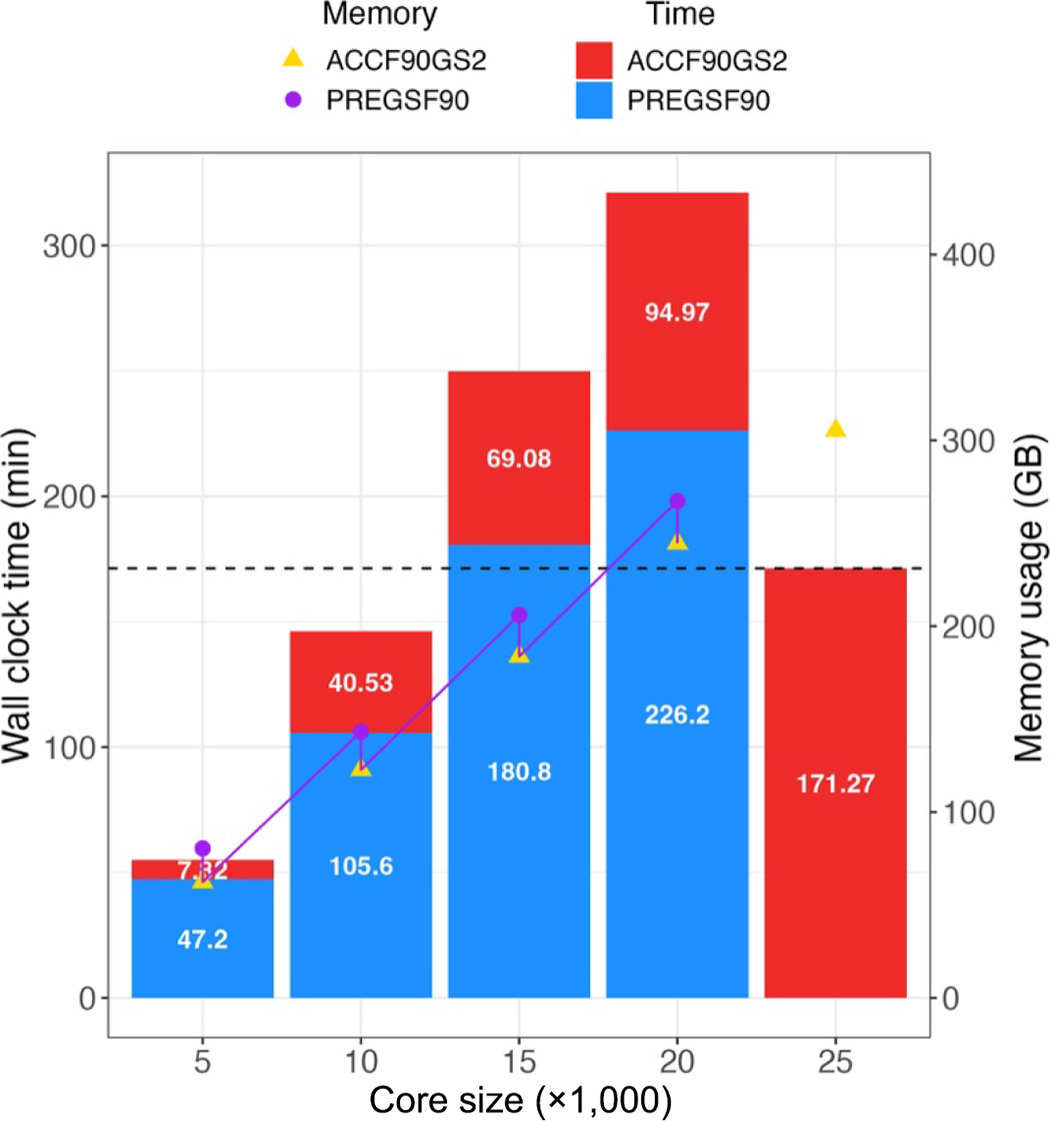
Wall clock time in minutes and memory requirements in GB for approximating reliabilities using different core sizes in $G_{\mathrm{APY}}^{-1}$ for Data1_C. Blue blocks (purple dots) refer to the elapsed time (memory use) for constructing $G_{\mathrm{APY}}^{-1},$ and red blocks (yellow triangles) represent the time (memory use) for obtaining reliability. The benchmark analysis with a core size of 25k does not include the requirements for estimating $G_{\mathrm{APY}}^{-1},$ as this matrix is calculated and stored beforehand during the breeding values estimation.

It is important to highlight that the impact of the core size reduction for reliability approximation is more pronounced in multiple-trait models. As Bermann et al. (2022a) pointed out, increasing the number of traits leads to an almost linear increase in the ACCF90GS2 computing time. Following this notion, we predicted the theoretical changes in computing time when approximating reliabilities for a 3-trait model with the same number of genotyped animals as in Data1_C. In this hypothetical scenario, approximating reliabilities would take 513.81 min with the benchmark core size, compared with only 70.66 min with the 5k core. Nevertheless, in multiple-trait analyses, further research is needed to quantify potential losses in approximation accuracy as well as the actual time requirements.

We investigated the impact of reducing the core set at a higher heritability (h^2^ = 0.30) in Data1_C (results not shown). As expected, this did not alter the computational demands of the algorithm. Instead, higher heritability mainly shifted the overall scale of the resulting reliability values. The pattern of changes was similar across genotyped animals, with the exception that animals retained in the core group were less affected by core-set reduction under higher heritability.

The comparison statistics between exact and approximated reliabilities calculated with smaller core sizes in $G_{APY}^{-1}$ for Data1_R are presented in Table 2. The intercept and slope of the regression of the exact reliability on the approximated reliabilities ranged from 0.20 to 0.22 and 0.78 to 0.80, respectively. Regarding the correlations and MD between exact and approximated reliabilities, we observed consistent magnitudes across all comparison scenarios. Correlations were almost always 0.91, and MD values were approximately 0.14.

**Table 2:** Correlation, intercept, slope, and mean difference (MD) between exact 25k-core reliabilities and the approximated reliabilities obtained using core sizes of 25k, 20k, 15k, 10k, and 5k in $G_{\mathrm{APY}}^{-1}$ for a reduced dataset (Data1_R)

Analysis	Group^1^	Correlation	Intercept	Slope	MD
25k	F-core	0.91	0.20	0.80	0.13
	N-core	0.91	0.20	0.80	0.13
20k	R-core	0.91	0.20	0.79	0.13
	N-core	0.91	0.20	0.80	0.13
	F-core	0.92	0.20	0.80	0.13
15k	R-core	0.91	0.20	0.79	0.13
	N-core	0.91	0.20	0.80	0.13
	F-core	0.92	0.20	0.80	0.13
10k	R-core	0.91	0.20	0.79	0.13
	N-core	0.91	0.21	0.80	0.14
	F-core	0.92	0.21	0.79	0.14
5k	R-core	0.91	0.20	0.79	0.13
	N-core	0.91	0.22	0.79	0.15
	F-core	0.92	0.22	0.78	0.15

1 N-core: animals consistently classified as noncore across all comparison scenarios; F-core: animals initially part of the benchmark (25k) core group but subsequently reclassified as noncore after core size reductions; R-core: animals from the benchmark core group that remained in the core after each reduction.

When the intercept is 0 and the slope is 1, the goodness of fit of the regression is optimal. Deviations from these values suggest that the estimates are over- or underestimated. Using the approximation algorithm with all evaluated core sizes, reliabilities in the reduced dataset were somewhat underestimated. Studies such as those by Ramos et al. (2024) and Ben Zaabza et al. (2022) also reported biased reliabilities when comparing approximation algorithms based on APY-ssGBLUP and SNP-BLUP models in beef and dairy cattle datasets. This lack of precision often stems from difficulties in accurately deriving effective record contributions using reverse reliability techniques, low heritabilities for certain traits, population structure, or an insufficient number of Monte Carlo samples in Bayesian regression-based models (Ben Zaabza et al., 2021).

Studies such as those by Abdollahi-Arpanahi et al. (2022) and Pocrnic et al. (2016) have demonstrated that decreasing the core size beyond the number needed to explain 98% of the variation in the GRM reduces the prediction accuracy of the breeding values. However, ours is the first study to evaluate how reducing the core size affects the reliability of breeding values within the APY framework. Taking the core size used in the estimation of GEBV as the ideal one for calculating reliabilities, which in this study was 25k, our findings indicate that for Data1_C, approximations obtained with core sizes smaller than 25k differed notably from one another and from the benchmark. In contrast, for Data1_R, reliability approximations obtained using cores explaining 90% and 99% of the genetic variation (i.e., 5k and 15k animals, respectively) were virtually indistinguishable. We hypothesize that this discrepancy may be influenced by the ratio of noncore to the core animals (noncore:core) in $G_{APY}^{-1}.$ In the benchmark analysis for Data1_C, this ratio was 64.2, whereas for Data1_R, it was only 0.4. The vast disparity in the noncore:core ratios between these 2 analyses likely explains why Data1_C results were more variable than those from Data1_R. This suggests that the larger the noncore:core ratio, the more susceptible the approximations are to fluctuations as the core size decreases compared with the ideal one. Nonetheless, because reliabilities are approximations and are not used directly for selection, reducing their precision slightly is unlikely to affect genetic evaluations or alter selection decisions.

We present practical strategies for reducing computing time when approximating reliabilities in ssGBLUP with APY. Our results demonstrate that minimizing the number of core animals can reduce the algorithm's computing time by up to 67% and memory usage by up to 53% for single-trait models involving $G_{APY}^{-1}$ matrices of up to 25,000 core animals. The time savings from core reductions become more meaningful as the number of genotyped animals and traits in the input data increases. However, decreasing the core size in $G_{APY}^{-1}$ may affect the precision of the approximated reliabilities depending on the extent of the reduction.

## References

[bib1] Abdollahi-Arpanahi R., Lourenco D., Misztal I. (2022). A comprehensive study on size and definition of the core group in the proven and young algorithm for single-step GBLUP. Genet. Sel. Evol..

[bib2] Ben Zaabza H., Mäntysaari E.A., Strandén I. (2021). Estimation of individual animal SNP-BLUP reliability using full Monte Carlo sampling. JDS Commun..

[bib3] Ben Zaabza H., Taskinen M., Mäntysaari E.A., Pitkänen T., Aamand G.P., Strandén I. (2022). Breeding value reliabilities for multiple-trait single-step genomic best linear unbiased predictor. J. Dairy Sci..

[bib4] Ben Zaabza H., Van Tassell C.P., Vandenplas J., VanRaden P., Liu Z., Eding H., McKay S., Haugaard K., Lidauer M.H., Mäntysaari E.A., Strandén I. (2023). Invited review: Reliability computation from the animal model era to the single-step genomic model era. J. Dairy Sci..

[bib5] Bermann M., Lourenco D.A.L., Misztal I. (2022). Efficient approximation of reliabilities for single-step genomic best linear unbiased predictor models with the algorithm for proven and young. J. Anim. Sci..

[bib6] Bermann M., Lourenco D., Forneris N.S., Legarra A., Misztal I. (2022). On the equivalence between marker effect models and breeding value models and direct genomic values with the algorithm for proven and young. Genet. Sel. Evol..

[bib7] Bermann M., Lourenco D., Cesarani A., Misztal I. (2022). Proceedings of the World Congress on Genetics Applied to Livestock Production, Rotterdam 2022.

[bib8] Brzáková M., Veselá Z., Vařeka J., Bauer J. (2023). Improving breeding value reliability with genomic data in breeding groups of Charolais. Genes (Basel).

[bib9] Gao H., Kudinov A.A., Taskinen M., Pitkänen T.J., Lidauer M.H., Mäntysaari E.A., Strandén I. (2023). A computationally efficient method for approximating reliabilities in large-scale single-step genomic prediction. Genet. Sel. Evol..

[bib10] Gonzalez-Peña D., Vukasinovic N., Brooker J.J., Przybyla C.A., DeNise S.K. (2019). Genomic evaluation for calf wellness traits in Holstein cattle. J. Dairy Sci..

[bib11] Gu M., Eisenstat S.C. (1996). Efficient algorithms for computing a strong rank-revealing QR factorization. SIAM J. Sci. Comput..

[bib12] Lourenco D., Tsuruta S., Aguilar I., Masuda Y., Bermann M., Legarra A., Misztal I., Veerkamp R.F., de Haas Y. (2022). Proceedings World Congress on Genetics Applied to Livestock Production.

[bib13] Misztal I., Legarra A., Aguilar I. (2014). Using recursion to compute the inverse of the genomic relationship matrix. J. Dairy Sci..

[bib14] Misztal I., Tsuruta S., Lourenco D.A.L., Masuda Y., Aguilar I., Legarra A., Vitezica Z. (2014). Manual for BLUPF90 family of programs. https://nce.ads.uga.edu/html/projects/programs/docs/blupf90_all8.pdf.

[bib15] Pocrnic I., Lourenco D.A., Masuda Y., Misztal I. (2016). Dimensionality of genomic information and performance of the algorithm for proven and young for different livestock species. Genet. Sel. Evol..

[bib16] Ramos P., Garcia A., Retallik K., Bermann M., Tsuruta S., Misztal I., Veroneze R., Lourenco D. (2024). Comparing algorithms to approximate accuracies for single-step genomic best linear unbiased predictor. J. Anim. Sci..

[bib17] Sarlos T. (2006). 2006 47th Annual IEEE Symposium on Foundations of Computer Science (FOCS'06).

[bib18] Van Vleck L.D. (1993). Variance of prediction error with mixed model equations when relationships are ignored. Theor. Appl. Genet..

